# Reduced Oxidative Stress Contributes to the Lipid Lowering Effects of Isoquercitrin in Free Fatty Acids Induced Hepatocytes

**DOI:** 10.1155/2014/313602

**Published:** 2014-10-22

**Authors:** Waseem Hassan, Gao Rongyin, Abdelkader Daoud, Lin Ding, Lulu Wang, Jun Liu, Jing Shang

**Affiliations:** National Center for Drug Screening and State Key Laboratory of Natural Medicines, China Pharmaceutical University, No. 24 Tongjiaxiang, Nanjing, Jiangsu 210009, China

## Abstract

Oxidative stress interferes with hepatic lipid metabolism at various levels ranging from benign lipid storage to so-called second hit of inflammation activation. Isoquercitrin (IQ) is widely present flavonoid but its effects on hepatic lipid metabolism remain unknown. We used free fatty acids (FFA) induced lipid overload and oxidative stress model in two types of liver cells and measured cell viability, intracellular lipids, and reactive oxygen species (ROS) within hepatocytes. In addition, Intracellular triglycerides (TG), superoxide dismutase (SOD), and malondialdehyde (MDA) were examined. A novel *in vitro* model was used to evaluate correlation between lipid lowering and antioxidative activities. Furthermore, 34 major cytokines and corresponding ROS levels were analyzed in FFA/LPS induced coculture model between hepatocytes and Kupffer cells. At molecular level AMPK pathway was elucidated. We showed that IQ attenuated FFA induced lipid overload and ROS within hepatocytes. Further, IQ reversed FFA induced increase in intracellular TG SOD and MDA. It was shown that antioxidative activity of IQ correlates with its lipid lowering potentials. IQ reversed major proinflammatory cytokines and oxidative stress in FFA/LPS induced coculture model. Finally, AMPK pathway was found responsible for metabolic benefits at molecular level. IQ strikingly manifests antioxidative and related lipid lowering activities in hepatocytes.

## 1. Introduction

Prevalent theories encompassing abnormal hepatic lipid metabolism have largely supported the role of oxidative stress at various levels. Unabated lipids overflow forces the mitochondrial dysfunctioning and subsequent production of higher ROS levels perturbs the normal functions of energy metabolism. Rising ROS levels accompany decline in oxidative defense mechanism to establish oxidative stress state. Number of theories ranging from implication of oxidative stress in so-called second-hit of NAFLD [1] to the involvement of ROS in enhancing lipid storage in first hit [2, 3] has put antioxidative pharmacological therapies to the major focus in treating metabolic abnormalities. Additionally, the proinflammatory characteristic of ROS to recruit or activate inflammatory cells within hepatocytes such as Kupffer cell and stellate cells to produce cytokines and certain degree of inflammation has been well established to further exacerbate the lipid metabolic state within liver [4, 5]. For example activation of Kupffer cells has been reported to generate the oxidative stress and series of proinflammatory cytokines [6], which in turn have been found to disrupt lipid metabolism. As the central role of oxidative stress in lipid metabolic abnormalities is rapidly gaining grounds, the antioxidant therapies are becoming ever important.

The hepatic lipid metabolism is a key source of energy for body and controls various metabolic states pathophysiologically. One of the most common reasons that can lead to hepatic lipid metabolic disturbance is lipid storage within hepatocytes. Lipid accumulation within hepatocytes primes the liver for metabolic complications which in turn can give rise to many chronic metabolic disease states like fatty liver [7], atherosclerosis [8], insulin resistance [9], and obesity [10]. Adenosine monophosphate-activated protein kinase (AMPK) is an energy sensitizer and master switch in various cellular metabolic pathways. AMPK has characterized its role in various aspects of lipid metabolism ranging from fat oxidation, mobilization, de novo synthesis, and lipid transport [11]. AMPK activation has shown profound effects in ameliorating lipid storage by inhibiting sterol regulatory element-binding proteins 1-c (SREBP-1c) and fatty acid synthase (FAS) to switch off its synthesis [12]. Further, it can serve as activator of peroxisome proliferator-activated receptor alpha (PPAR-*α*) upon its phosphorylation [13], which is an important player in enhancing fatty acid oxidation through regulating various fat transport proteins including Carnitine palmitoyltransferase-1 (CPT-1). Although oxidative stress is speculated to inhibit the AMPK activation but their exact relationship remained obscure by conflicting reports.

Flavonoids are the class of ubiquitously present plant-derived polyphenolic compounds with widely recognized anti-inflammatory [14, 15], antimicrobial [16], anticancer [17], and antiallergic [18] activities. Isoquercitrin (IQ), a flavonoid, is a widely existing quercetin glycoside present in numerous nutritional sources of vegetables and fruits species [19]. It has shown to possess robust anti-oxidative activity among other useful medicinal potentialities [20, 21]. Recently, IQ has emerged as a likely prospect in metabolic disorders. Various studies in recent past have highlighted the beneficial effects of IQ in insulin resistance [22] and fat oxidation [23] in both* in vivo* and* in vitro* models. Moreover, IQ has previously manifested its inhibitory activities on adipocytes differentiation [24]. Taking in account the beneficial effects of IQ on fat metabolism and oxidative stress, we sought to investigate potential lipid lowering and antioxidative activity of IQ on free fatty acid (FFA) induced hepatocytes.

In the present study, we aim to scrutinize the antioxidative, anti-inflammatory, and lipid lowering activities of IQ in FFA activated primary rat hepatocytes and rat liver cell line BRL-3A. We also aim to investigate the relationship between these activities, responsible for exerting the metabolic benefits within hepatocytes.

## 2. Methods

### 2.1. Reagents

Oil red dye, hydrogen peroxide (H_2_O_2_), and N-acetyl cysteine (NAC) were purchased from Sigma-Aldrich (St Louis, MO, USA). FFA free bovine serum albumin (BSA) was obtained from Merck (Germany). Detection kits for SOD and MDA were from NJJCBIO Inc. (Nanjing, China), while commercial detection kit for triacylglycerides (TG) was taken from Beijing BHKT (Beijing, China). ROS detection kit was used from Beyotime (Haimen, Jiangsu, China). The Takara quantitative RT-PCR kit and SYBR Green Premix Ex Taq were products of Takara Biomedicals Inc. (Shiga, Japan). The primary and secondary antibodies used for Western blot (AMPK, AMPK-p, p-ACC, and *β*-actin) were purchased from Abcam (Cambridge, UK). Compounds C and AICAR were obtained from Tocris Bioscience (Minneapolis, MN, USA). Cytokine array kit was purchased from RayBiotech, Inc. (Norcross, Ga.) All other laboratory chemicals were of the highest purity from commercial suppliers.

### 2.2. Preparation of FFA and FFA/LPS Combination

The mixture of oleate and palmitate (2 : 1) was used to induce the hepatic lipid accumulation as mentioned elsewhere with minor changes [25]. In brief, 100 mM of oleate and palmitate was dissolved in DMSO and the product was filtered. 5% BSA (Merck, Germany) was prepared by dissolving it in culture medium. The final concentration of BSA was kept 1% in all experiments to mimic the physiological ratio between bound and unbound FFA in media [26]. Combination of lipopolysaccharides (LPS) (10 ng/mL) and free fatty acids (FFA) (0.5 mM) prepared in 1% bovine serum albumin (BSA) was used to induce the coculture between hepatocytes and Kupffer cells.

### 2.3. Isolation of Primary Hepatocytes and Kupffer Cells

Isolation of rat hepatocytes was performed as mentioned elsewhere [27] with some modification. A two-step perfusion method was implied to obtain the primary rat hepatocytes. Shortly, perfusion buffer-1 (EDTA to 0.5 mM; HEPES to 25 mM) was prepared in Hanks balance salt solution (HBSS) without Ca^2+^ and Mg^2+^ while perfusion buffer-2 (Collagenase-II) was prepared in medium. The hepatocytes were isolated from SD rats and kept on fast for 12 hours before experiment. On the day of experiment, rats were anaesthetized and perfused with perfusion buffers at a constant speed. After meshy appearance, liver was excised and transferred to cold medium. Hepatocytes were shredded, filtered, and centrifuged at 50 g for 3 minutes at 4°C. The pellets predominantly contain the hepatocytes population, which was further purified by 90% percoll gradient. Final population was counted by trypan blue. Hepatocytes were identified by using anti-CK18 antibody (1 : 200, abcam). The supernatant which largely possesses the nonparenchymal cells like KCs transferred to separate tube for further processing to isolate KCs. Counted hepatocytes were cultured in well plates and cell viability was determined periodically.

KCs were isolated from normal rats as mentioned elsewhere [27] with some modification. Briefly, the supernatants obtained as described above were centrifuged at 450 ×g for 10 minutes at 4°C to sediment the nonparenchymal cells. Pellets were collected and washed with PBS twice. Two-step percoll (Biosharp, China) gradient cushion (25%/50% (vol/vol)) was used to centrifuge the cells at 1000 ×g for 10 min at 4°C with the brake option off. The upper layers were discarded, and the layer between 25% and 50% percoll cushion was collected without mixing. The layer was transferred to fresh tube, washed with DMEM containing 10% FBS, and plated into six well plates. Nonadherent cells were removed by washing after two hours of incubation at standard conditions. KCs were identified by their typical morphological features.

### 2.4. Coculture Model

Hepatocytes in 6-well cell culture inserts (Millipore; cat. number PIHT30R48) were cocultured with KCs in 6-well plates at approximately 1 : 4 ratio. Both types of cells were cocultured in Williams E (Invitrogen, CA, USA) medium supplemented with 100 units/mL penicillin and 100 mg/mL streptomycin. After an initial attachment period of 4 h, fresh medium was administered in each culture well containing hepatocytes and KCs. Lipopolysaccharides (LPS) (10 ng/mL) and free fatty acids (FFA) (0.5 mM) prepared in 1% bovine serum albumin (BSA) were used to induce the KCs, while simultaneously hepatocytes were cotreated with IQ-H (50 *μ*M) for 24 hours in coculture model. Afterwards, conditioned media from hepatocytes were used to observe the changes in cytokines profile.

### 2.5. Cell Culture and Primary Cell Treatment

Normal rat liver cell line BRL-3A was obtained from ATCC, (Manassas, VA, USA) and cultured in DMEM (Invitrogen) containing 10% (v/v) foetal bovine serum, 5 U/mL penicillin and 50 *μ*g/mL streptomycin at 37°C in a humidified atmosphere. Cells were cultured in 96-well plates and 6-well plates for different experiments. Primary hepatocytes were cultured in William E (Invitrogen) containing insulin to 100 nM, dexamethasone to 100 nM, penicillin to 100 IU/mL, and streptomycin to 100 mg/mL.

### 2.6. Cell Viability Assay

Cell viability was assessed by 3-(4,5-dimethyl-2-thiazolyl)-2,5-diphenyltetrazolium bromide (MTT) colorimetric assay. MTT is converted from yellow to purple formazan by cellular dehydrogenases after taken up by live cells. Assay was performed as mentioned elsewhere [28]. The resultant absorbance was measured with plate-reader at 490 nm test wavelength using reference wave length of 650 nm.

### 2.7. Oil Red O Staining

Oil red O staining was performed as mentioned before [29] with few adjustments. In brief, treated BRL-3A cells and primary rat hepatocytes were washed thrice with PBS then fixed with 4% paraformaldehyde for 30 minutes. Cells were incubated with 60% paraformaldehyde and stained with oil red O for 15 minutes and 30 minutes for BRL-3A and primary cells, respectively. Cells were washed and extracted using 100% isopropanol. Pictures were taken with Olympus IX70 microscope in combination with a MicroFire digital camera at ×400 magnifications. OD was measured at 500 nm wave length.

### 2.8. Flow Cytometry and Fluorescence Microscopy

The BRL-3A cells or primary rat hepatocytes were cultured in 6-well plates and treated with IQ, simvastatin, and/or 1 mM FFA for required periods of time. The levels of ROS are measured with flow cytometric method and fluorescence microscopy. For flow cytometry, cells were harvested, washed, and incubated with 10 *μ*M H2DCF-DA for 30 minutes before taking it for analysis. For fluorescence microscopy, cells were washed and incubated with 10 *μ*M H2DCF-DA for 30 minutes. Digital photographs were captured with Olympus IX70 microscope attached with charged-coupling device camera. Fluorescence was measured at 490 nm excitation and 535 nm emission wave lengths. Fluorescence images were quantified with fluorescent imaging software (ImageJ) and expressed as percentage controls in comparisons. NAC was used as a ROS scavenger in different experiments. p-AMPK was analyzed by using FITC linked secondary antibody.

### 2.9. Measurement of TG, SOD, and MDA

Analysis of TG, SOD, and MDA was carried out by commercially available colorimetric kits as mentioned in the protocols. Cells were cultured in 6-well plates and cotreated with or without simvastatin, IQ, and/or 1 mM FFA. TG, SOD, and MDA were measured at 500 nm, 450 nm, and 530 nm, respectively. BCA test was used for quantifying proteins.

### 2.10. Cytokine Array

Supernatant from coculture was collected and analyzed with cytokine array by using rat cytokine antibody array (RayBiotech, Inc., Norcross, Ga.) according to the manufacturer's instructions. Briefly, cytokine array membranes were blocked in 2 mL of 1×blocking buffer for 30 min and incubated with 1 mL of samples at 4°C overnight. Samples were removed from each box of incubation tray, and membranes were washed thrice with 2 mL of 1×wash buffer I, followed by two washes with 2 mL of 1×wash buffer II at room temperature with gentle rocking. Afterwards, membranes were incubated in 1 : 250 diluted biotin-conjugated primary antibodies at 4°C overnight and washed with buffers I and II as mentioned above. Membranes were then incubated in 1 : 1,000-diluted horseradish peroxidase-conjugated streptavidin at room temperature for 2 hours. Membranes were washed as described above and exposed to a peroxidase substrate (detection buffers C and D; RayBiotech, Inc.) for 5 min in the dark before imaging. Membranes were exposed to X-ray film (Kodak X-OMAT AR film) within 15 min of exposure to the substrate. Signal intensities were obtained by Bio-Rad Imaging System and analyzed with Quantity One software (Bio-Rad). Biotin-conjugated immunoglobulin G served as a positive control at four spots, to normalize and compare the different membranes.

### 2.11. Quantitative Real-Time PCR

Total RNA was extracted from primary cells using TRIZOL reagent (Gibco-BRL, USA). First strand cDNA was synthesized with PrimeScript RT Master Mix (Takara, Japan) according to the manufacturer's instructions. The quantitative real-time PCR was performed on an iQ5 multicolor real-time PCR detection system (Bio-Rad, Hercules, Calif., USA) by using SYBR Premix Ex TaqTM (Takara, Japan) according to the manufacturer's instructions. To investigate the effects of IQ on lipid metabolism, the mRNA expression of SREBP-1c, FAS, and CPT-1 was examined. Primer sequences were as follows: SREBP-1c—(5′-ACAGCACAGCA ACCAG AAACTC-3′) and (5′-TTCATGCCCTCCATAGACACAT-3′); FAS (5′-TTGGCTTAG TGAT TGCATCTCGT-3′) and (5′-CAGGGTCTCTGTCCTCCTTTTGT-3′); CPT-1 (5′-TCAGAGGATGGACACTGTAAAGGAG-3′) and (5′-CCGAAAGAGTCAAATGGGAAGG-3′). Real-time PCR conditions were 1 cycle of 2 min at 50°C, 95°C for 10 min, followed by 40 cycles of 95°C cDNA denaturation for 20 s, 60°C primer annealing for 30 s, and 72°C extension for 30 s. The expression levels of each gene were normalized against GAPDH using the comparative 2^−ΔΔCT^ method and the results were from three independent experiments according to the manufacturer's protocols [30].

### 2.12. Protein Extraction and Western Blotting

After treatment proteins were extracted and subjected to Western blotting. Briefly, proteins were scraped with the 100 *μ*L protein lysis buffer containing 0.5 mM PMSF. After centrifugation at 14,000 g at 400°C for 20 min, the cellular proteins were transferred to the fresh eppendorf tube. The proteins samples were heated at 100°C for 5 minutes to denature the proteins. Protein concentrations of the protein samples were determined by use of BCA. 10% SDS-PAGE was used to transfer the proteins onto PVDF membrane. Then membranes were incubated with the appropriate concentration of primary antibody (abcam). Protein bands were developed using chemiluminescence detection reagents before being visualized and captured with the ChemiDoc imaging system (Bio-Rad Laboratories, Hercules, CA, USA).

### 2.13. Statistical Analysis

Statistical analysis was achieved by using graph pad prism Version 5.0c (GraphPad Software). Quantitative data are expressed as mean ± SD. Data were analyzed, as relevant, by unpaired two-tailed Student's *t*-test or by one-way ANOVA with Turey's post hoc test. *P* value of 0.05 was accepted as statistically significant.

## 3. Results

### 3.1. Effects of IQ, FFA, and LPS on Cell Viability in BRL-3A Cells and Primary Rat Hepatocytes

**Figure 1:**
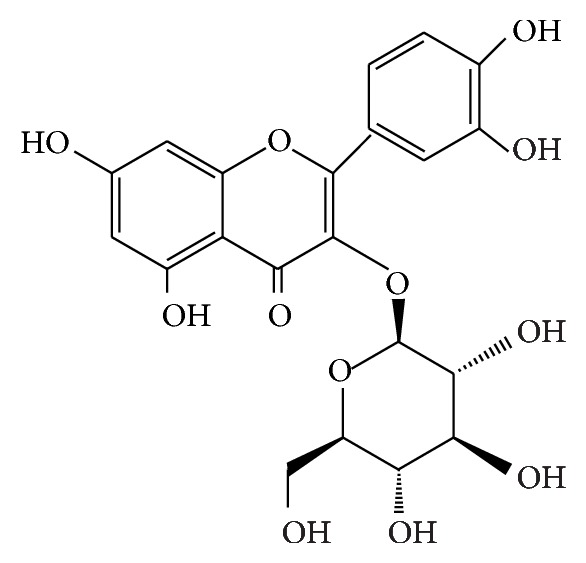
Chemical structure of isoquercitrin (IQ).

**Figure 2:**
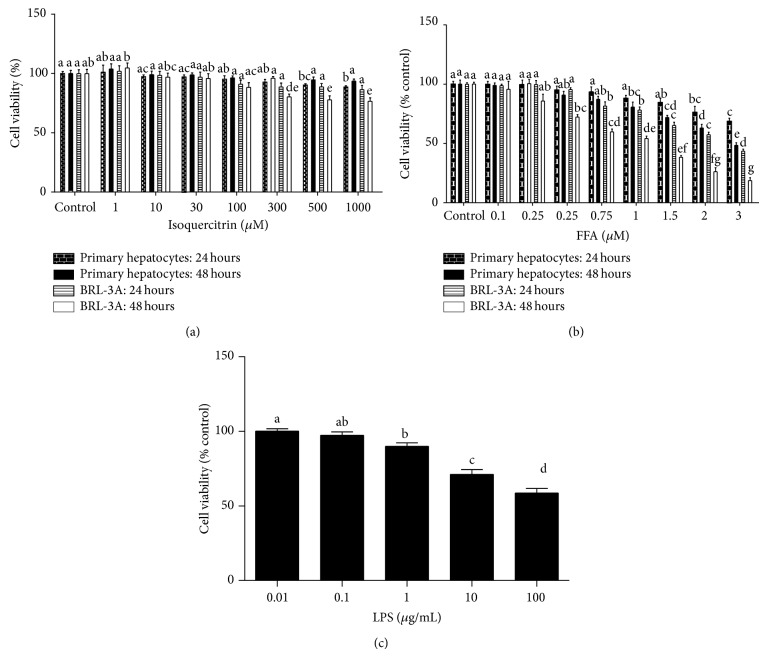
Cell viability assay on BRL-3A cells and primary rat hepatocytes. Cells were cultured in 96-well plates and treated with different doses of isoquercitrin (a) and FFA (b) for 24 hours and 48 hours. (c) Primary hepatocytes were cultured in 96-well plates and treated with various LPS doses for 12 hours. After the required period of time cells were administered with MTT for 3 hours before dissolving it with organic solvent. The data represents ±SD. *P* < 0.05 was considered as statistically significant. Different superscripts letters demonstrate the significant statistical difference as calculated by ANOVA followed by Tukey's test.

IQ (Figure 1) has no substantial effects on cell viability of either BRL-3A cell line or primary rat hepatocytes (Figure 2(a)) up to the dose of 1000 *μ*M. None of the group was less than 75% viable after maximum of 48 hours of treatment in both types of cells. In addition, FFA treatment (0.1 mM–3 mM) decreased the cell viability in both types of liver cells (Figure 2(b)). However BRL-3A cells were more sensitive to FFA both time and dose dependently as compared to primary rat hepatocytes (Figure 2(b)). Cell viability in LPS induced primary rat hepatocytes is demonstrated in Figure 2(c).

### 3.2. IQ Lowers the FFA Induced Lipid Accumulation

**Figure 3:**
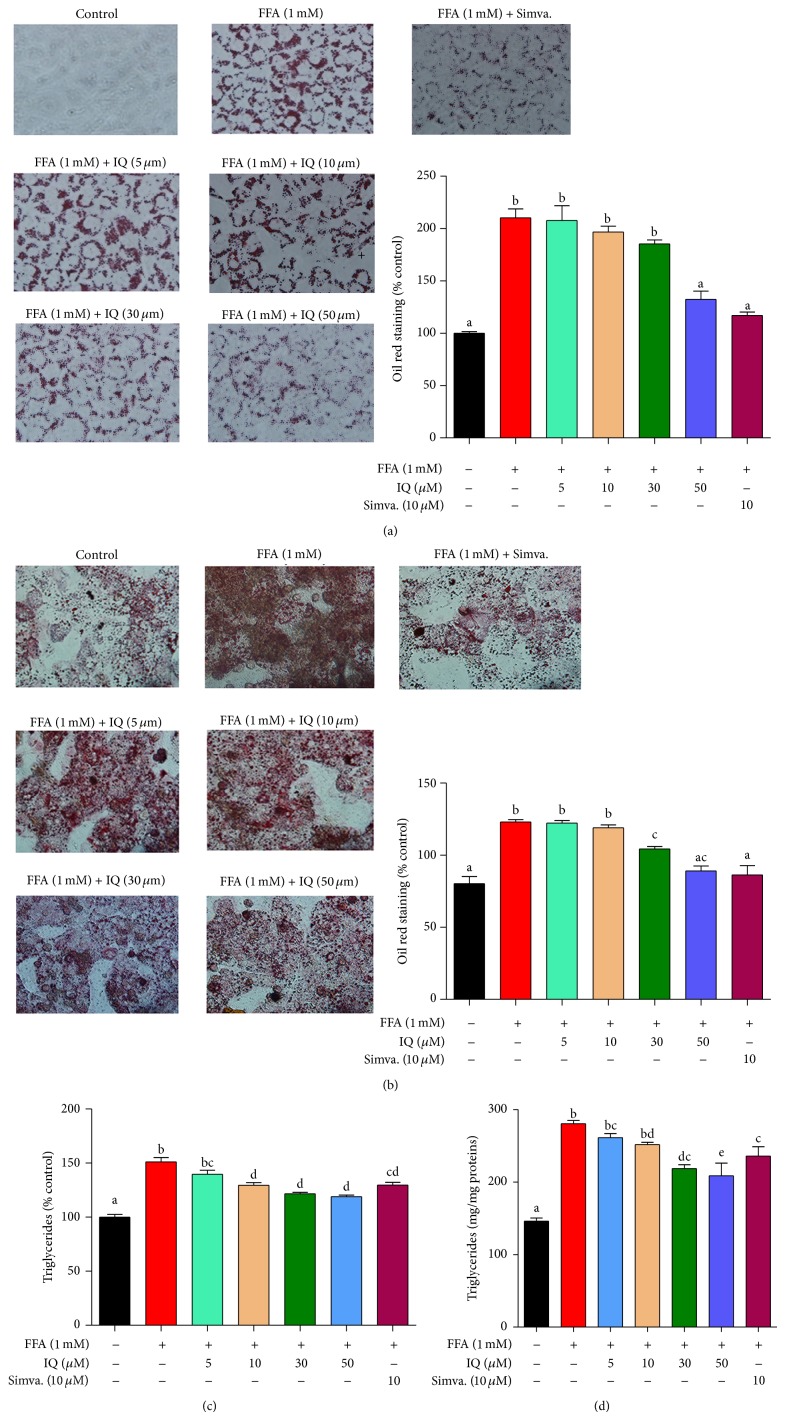
Illustrates the effects of isoquercitrin at different doses on hepatic lipid accumulation and intracellular triglycerides (TG). BRL-3A cells ((a) and (c)) and primary rat hepatocytes ((b) and (d)) were treated with 1 mM FFA and/or isoquercitrin for 24 hours. After the treatment duration cells were washed, fixed, and stained with oil red o dye. The images of cells were captured by microscope at ×400 of original magnification. Intracellular TG was measured by commercially available kits. Proteins were calculated by BCA method. Values for primary rat hepatocytes were expressed in mg/mg proteins, while values for BRL-3A cells were indicated in % control. The data represents ±SD. *P* < 0.05 was considered as statistically significant. Different superscripts letters demonstrate the significant statistical difference as calculated by ANOVA followed by Tukey's test.

As it is illustrated in Figure 3(a), IQ dose dependently reduced the FFA (1 mM) induced lipid accumulation in BRL-3A cell line. Similar effects were observed in primary rat hepatocytes in which IQ dose proportionally attenuated the FFA induced lipid storage (Figure 3(b)). At 50 *μ*M, IQ significantly reduced the lipid accumulation in both types of liver cells.

To overturn the chances that IQ may have reacted with FFA before the hepatocytic uptake and thus showed its enhanced lipid lowering activity, we first treated all groups with FFA for 24 hours and then administered IQ for another 24 hours. As it is shown in Figure 6(a), IQ prevented the prior FFA induced lipid overload in hepatocytes with dose dependency as compared to group treated with FFA (Figure 6(a)).

### 3.3. IQ Retards FFA Stimulated Intracellular Triglycerides (TG)

The storage of TG is a hallmark of hepatic lipid disorders. Expectedly, IQ also diminished the intracellular TG levels in BRL-3A (Figure 3(c)) and primary rat hepatocytes (Figure 3(d)) as compared to the FFA administered group. Moreover, substantial decrease of TG at higher IQ doses (50 *μ*M) correlated with the effects of IQ (50 *μ*M) on total lipids (Figures 3(a) and 3(b)) in both types of cells.

### 3.4. IQ Inhibits FFA Induced ROS Generation

**Figure 4:**
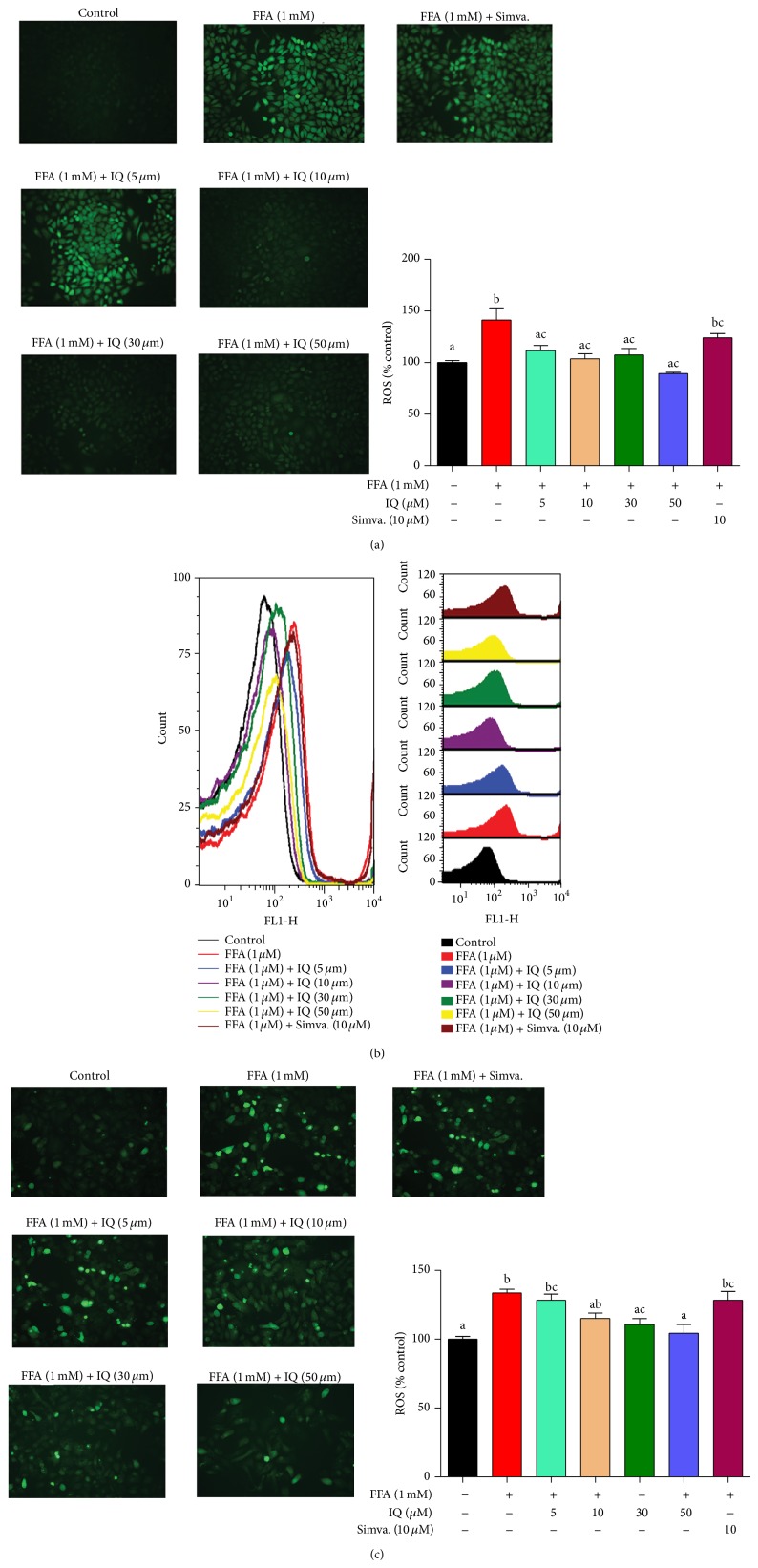
Effects of IQ on ROS in FFA induced primary rat hepatocytes and BRL-3A cells. (a) shows the pictographs of effects of IQ on BRL-3A cells. Magnification ×200 (b) illustrates the histograms from flow cytometry showing effects of IQ on primary rat hepatocytes. (c) demonstrates the pictographs of primary rat hepatocytes exhibiting effects of IQ. Magnification; ×200. The data represents ±SD. *P* < 0.05 was considered as statistically significant. Different superscripts letters demonstrate the significant statistical difference as calculated by ANOVA followed by turkey's test.

Figure 4(a) shows that IQ drastically decreased the cellular ROS levels in BRL-3A hepatocytes treated with different doses of IQ with or without 1 mM FFA. Comparable confirmations were found in primary rat hepatocytes as well (Figures 4(b) and 4(c)).

Similarly, to reduce the chances of any cross-reaction between FFA and IQ before the hepatocytic uptake and hence affecting the antioxidative potential within hepatocytes, we employed the prior administration of FFA for 24 hours and then treated with IQ for another 24 hours. As it can be observed in Figure 6(b), IQ significantly reversed the FFA induced ROS in primary rat hepatocytes in prefat overload model as well.

### 3.5. The Effects of IQ on Antioxidant Enzyme (SOD) and Lipid Peroxidation Products (MDA)

**Figure 5:**
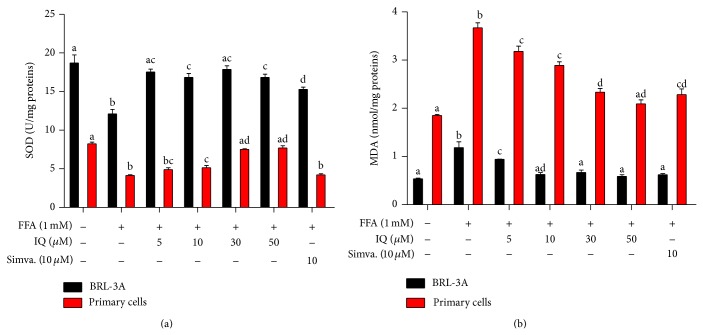
Actions of IQ on FFA induced SOD and MDA levels in BRL-3A cells and primary rat hepatocytes. (a) Graph represents the SOD value in BRL-3A cells and primary rat hepatocytes. (b) illustrates the MDA measurements in BRL-3A cells and primary rat hepatocytes. Values for SOD and MDA are expressed in U/mg-proteins and nmol/mg-protein, respectively. The data represents ±SD. *P* < 0.05 was considered as statistically significant. Different superscripts letters demonstrate the significant statistical difference as calculated by ANOVA followed by Tukey's test.

**Figure 6:**
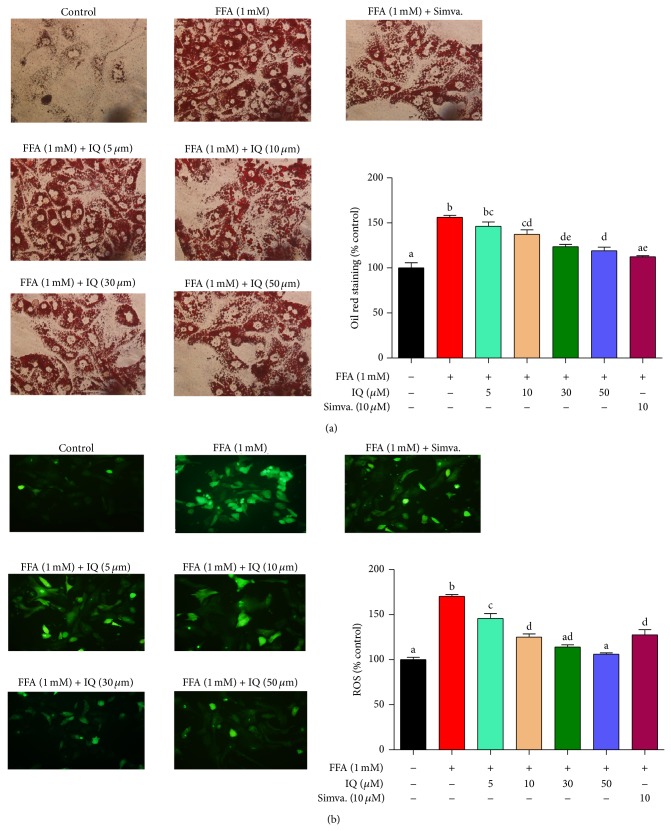
Effects of IQ on intracellular lipid accumulation and ROS levels. Primary rat hepatocytes were treated with FFA for 24 hours first and then treated with IQ for another 24 hours. Lipid accumulation was determined by oil red staining while ROS was analyzed with H2DCF-DA by the method described in material and methods. (a) shows the effects IQ on lipid accumulation; (b) displays the effects of IQ on ROS. The data represents ±SD. *P* < 0.05 was considered as statistically significant. Different superscripts letters demonstrate the significant statistical difference as calculated by ANOVA followed by Tukey's test.

We further investigated the effects of IQ on intracellular SOD levels. In addition, lipid peroxidation product, MDA, involved in lipid degradation was also analyzed. As demonstrated in Figure 5(a), FFA notably lowered the SOD levels in primary rat hepatocytes and treatment with IQ considerably enhanced the SOD levels in dose dependent manner. Although similar effects were observed in BRL-3A cells (Figure 5(a)) but the effects of IQ were less dose dependent as IQ enhanced the FFA induced SOD suppression at much lower doses (10 *μ*M) and effects were relatively maintained at higher doses (50 *μ*M) as well. Additionally, IQ reversed the MDA augmented by FFA (1 mM) in primary rat hepatocytes and BRL-3A cells (Figure 5(b)).

### 3.6. Lipid Lowering Effects of IQ Coexists with Its Antioxidative Potential

**Figure 7:**
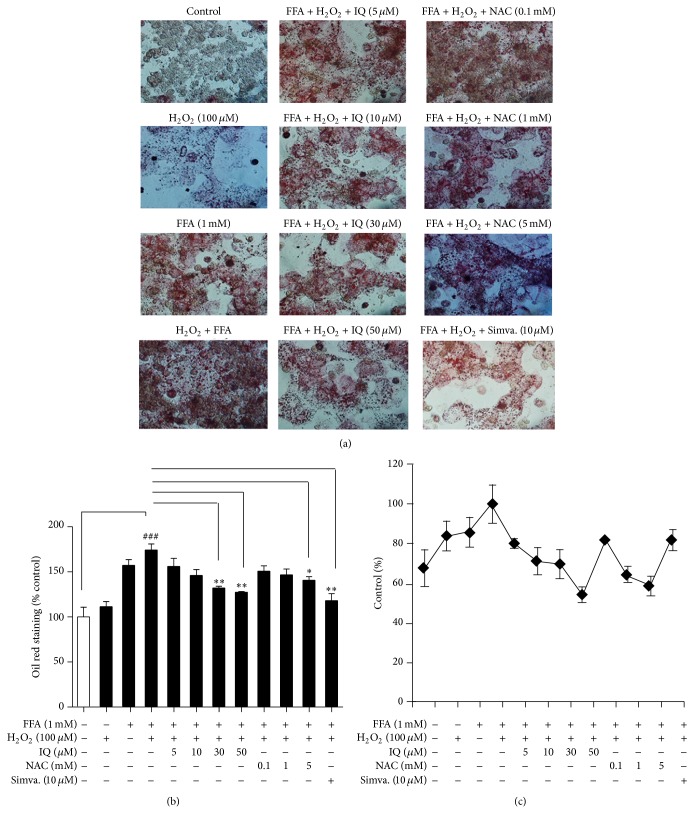
Correlation between reactive oxygen species (ROS) and hepatic lipid accumulation. Primary rat hepatocytes were first treated with several IQ does, simvastatin (10 *μ*M), or N-acetyl cysteine for 6 hours to analyze its preventive actions. After 6 hours, cells were left untreated (control and H_2_O_2_ group) or further treated with 1 mM FFA (FFA) for 14 hours to induce the lipid overload state within hepatocytes. Afterwards, cells were additionally treated with H_2_O_2_ (100 *μ*M) or left untreated (FFA and control group) for promoting the lipid overload state with exaggerated induced state of oxidative stress. Cells were analyzed for lipid accumulation (a) and (b) by oil red staining method as described above. Pictures (a) were captured at ×200 magnifications and quantification (b) was expressed as % control. Alternatively, cells were analyzed for ROS analysis to draw corelation (c) by H2DCF-DA method as described above. The data represents ±SD. ^*^ *P* < 0.05 versus model; ^**^ *P* < 0.01 versus model; ^***^ *P* < 0.001 versus model; ^##^ *P* < 0.01 versus model; ^###^ *P* < 0.001 versus model.

We sought to induce the primary rat hepatocytes with hydrogen peroxide (H_2_O_2_) in addition to FFA (1 mM) to generate the high oxidative stress conditions. Primary rat hepatocytes were treated with H_2_O_2_ (100 *μ*M) and FFA (1 mM) alone or combined (H_2_O_2_ and FFA), to evaluate whether increased ROS challenge has any effects on the lipid accumulation state within hepatocytes. Furthermore, cells were cotreated with various IQ and NAC doses. As it is displayed in Figure 7(a), FFA (1 mM) combined with H_2_O_2_ (100 *μ*M) markedly aggravated the hepatic lipid storage, while the condition was reversed dose dependently by IQ. NAC reversed the exaggerated oxidative stress challenge (FFA + H_2_O_2_) but to lesser degree as compared to IQ. Analysis of Figure 7(c) shows that IQ reduced the cellular ROS levels and these effects were matched with their lipid lowering activity.

### 3.7. Effects of IQ on Cytokines Profile and ROS in Coculture Model between KCs and HCs

We observed the broad range of cytokines in coculture model between KCs and HCs.

**Figure 8:**
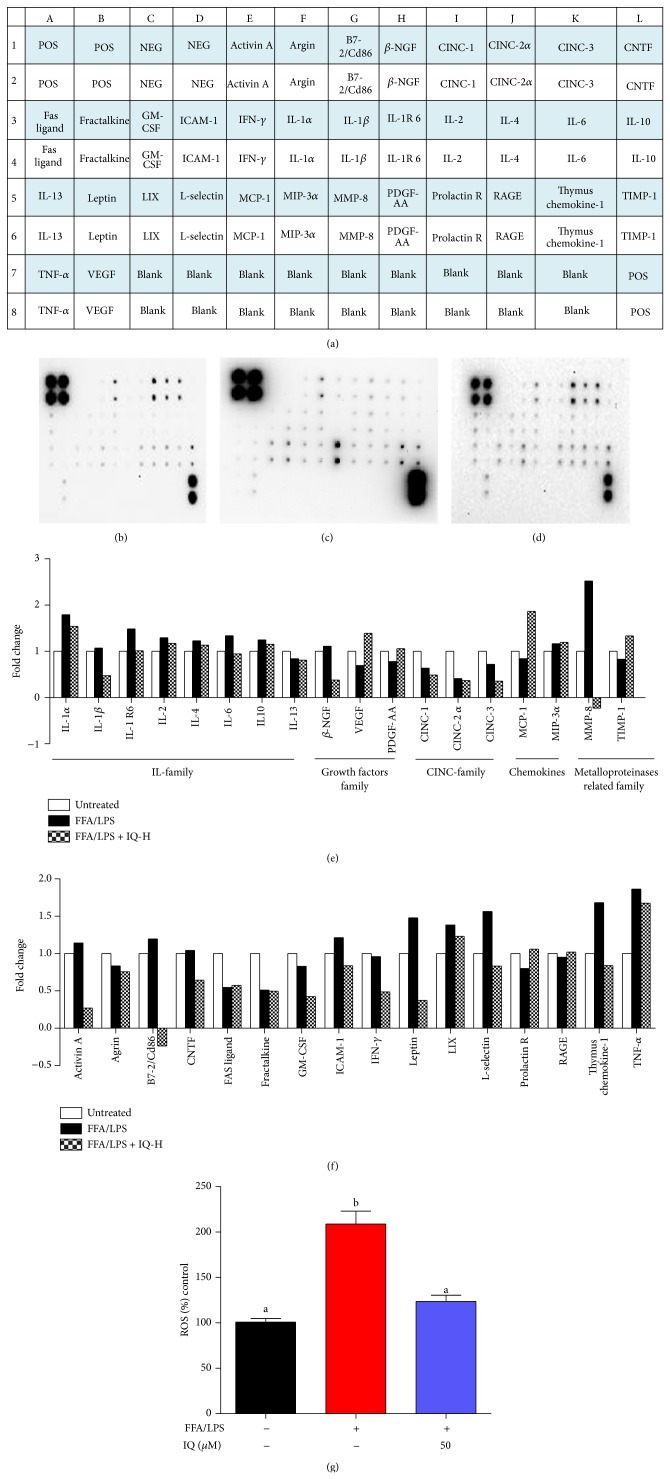
Activities of IQ on cytokines profile. Primary rat hepatocytes were cocultured with Kupffer cells and induced with FFA/LPS or left untreated for 24 hours. Cells were cotreated with IQ-H (50 *μ*M) for 24 hours. Afterwards, supernatants were collected and subjected to cytokine antibody array. (a) shows the array map. Each cytokine is represented as twin blots on array map. (b) untreated membrane; (c) FFA/LPS induced membrane; (d) IQ-H cotreated membrane. (e) illustrates the graphical representation of IL family cytokine, CINC family, growth factors, macrophage related cytokines, and MMP related cytokines. (f) analyses the changes in various other potentially important cytokines. Array blots are expressed as fold change. Average net optical intensities from each group for single cytokine were used to measure fold changes using ND group as reference. (g) measured the ROS in above-described coculture model. The data represents ±SD. *P* < 0.05 was considered as statistically significant. Different superscripts letters demonstrate the significant statistical difference as calculated by ANOVA followed by Tukey's test. POS, positive; NEG, negative; beta-NGF, nerve growth beta; CINC, cytokine-induced neutrophil chemoattractant; CNTF, ciliary neurotrophic factor; GM-CSF, granulocyte macrophage colony-stimulating factor; ICAM, intercellular adhesion molecule; IFN-gamma, interferon gamma; IL, interleukin; IL-1 R6, interleukin-1 receptor 6; LIX, C-X-C motif chemokine 5; MCP-1, monocyte chemoattractant protein-1; MIP-3, macrophage inflammatory protein-3; MMP-8, matrix metalloproteinase; PDGF-AA, platelet derived growth factor-AA; prolactin R, prolactin receptor; RAGE, advanced glycosylation end product receptor; TIMP-1, tissue inhibitor of metalloproteinases-1; TNF-alpha, tumour necrosis factor-alpha; VEGF, vascular endothelial growth factor.

Serum cytokines and growth factors are increasingly recognized as the markers for fat and LPS induced inflammatory state that, in turn, are reported to raise the oxidative stress within cells. We opted to perform cytokine array (Figure 8) to scrutinize the gross changes in cytokine profile. IQ treated groups in KC and HC coculture (Figure 8(d)) showed considerable decrease in proinflammatory cytokines profile as compared to untreated (Figure 4(b)) and FFA/LPS treated group (Figure 4(c)). IL family, CINC family, growth factors, macrophages related cytokines, and MMP family showed significant changes (Figure 8(e)) in FFA/LPS induced cells while IQ (50 *μ*M) considerably reversed these modifications. Other major cytokines affected by IQ and HFD are provided in Figure 8(f). Interestingly, levels of ROS also shifted to higher levels as the proinflammatory cytokines increased in coculture supernatant (Figure 8(g)).

### 3.8. Isoquercitrin Induces the AMPK Pathway

**Figure 9:**
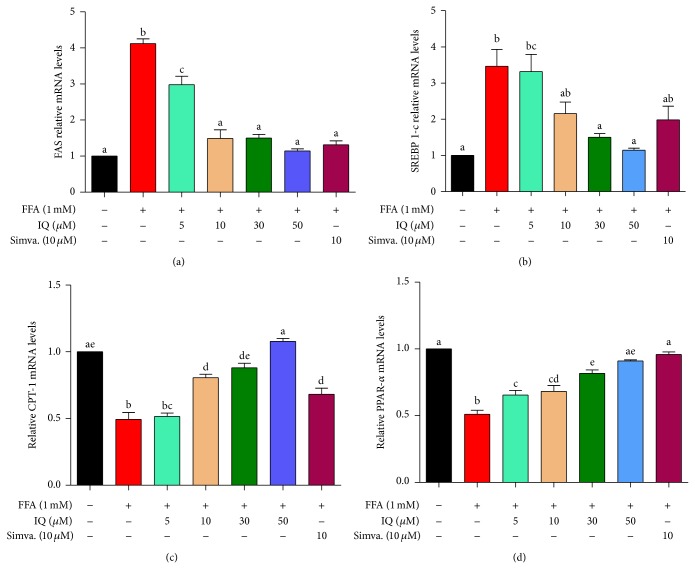
Effect of IQ on the expression of the lipogenic and lipolytic genes, FAS (a), SREBP-1c (b), CPT-1 (c), and PPAR-*α* (d) in primary rat hepatocytes. Gene expressions of SREBP-1c and FAS were decreased while CPT-1 and PPAR-*α* were increased by treatment with different doses of IQ under FFA challenge. Gene expression was quantified by qRT-PCR. The results represent the average of 3 independent experiments performed in triplicate. The relative expression level is presented as the fold increase compared with the control group. The data represents ±SD. *P* < 0.05 was considered as statistically significant. Different superscripts letters demonstrate the significant statistical difference as calculated by ANOVA followed by Tukey's test.

**Figure 10:**
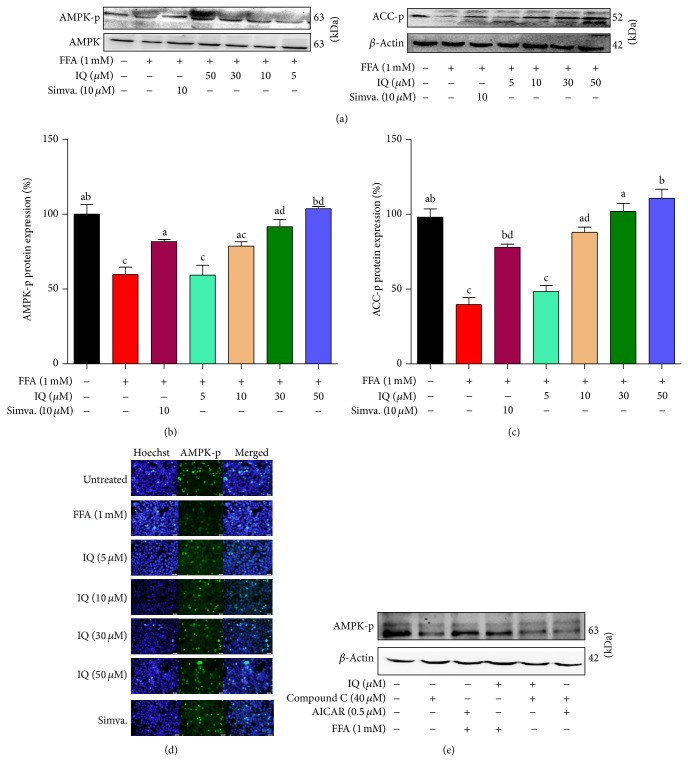
Validation for the effects of IQ in regulating AMPK pathway. (a) Representative western blots of AMPK, AMPK-p (and (b)), and ACC-p (and (c)). Primary rat hepatocytes were treated with FFA to induce the cells and coadministered with IQ or simvastatin for 24 hours to observe the changes in protein expression. (d) illustrates the immunofluorescence pictographs for p-AMPK expression in FFA induced primary rat hepatocytes. (e) shows the actions of IQ and AICAR on compound C blocked primary rat hepatocytes. Cells were treated with compounds C, IQ, and AICAR alone with or without the presence of FFA. Alternatively, IQ and AICAR were coadministered with compound C to draw comparisons. The data represents ±SD. *P* < 0.05 was considered as statistically significant. Different superscripts letters demonstrate the significant statistical difference as calculated by ANOVA followed by Tukey's test.

AMPK pathway has a central role in fatty acid oxidation, lipid and lipoprotein metabolism, inflammatory responses, and oxidative stress. mRNA expressions of AMPK dependent downstream genes FAS (Figure 9(a)), SREBP-1c (Figure 9(b)), CPT-1 (Figure 9(c)), and PPAR-*α* (Figure 9(d)) were downregulated by FFA, while IQ notably enhanced the mRNA expression of these key metabolic and oxidative genes. Furthermore, proteins expressions of both AMPK-p (Figures 10(a) and 10(b)) and ACC-p (Figures 10(a) and 10(c)) were downregulated in a group administered with FFA alone. IQ dose dependently raised the proteins expression of ACC-p and phosphorylated the AMPK. Our immunofluorescence experiments confirmed these findings in primary hepatocytes (Figure 10(d)). To confirm our finding on AMPK pathway, we selected compound C, a reversible AMPK inhibitor, and 5-aminoimidazole-4-carboxamide ribonucleotide (AICAR), AMPK activator. As it can be observed in Figure 10(e), the effects of IQ and AICAR on AMPK phosphorylation were blocked with the administration of compound C. AICAR and IQ administered alone were able to sufficiently phosphorylate the AMPK.

## 4. Discussion

In the present study, we provide the first verification for the protective actions of IQ on FFA induced oxidative stress and hepatic lipid accumulation in two types of liver cells. We schematically proved that IQ has beneficial effects in ameliorating hepatocytes lipid storage and its remarkable antioxidative potential contributes at least partly to regulate these effects. Furthermore, at molecular level, AMPK and its downstream dependent genes were found to be activated by IQ. In addition, we analyzed the effects of IQ on 34 major cytokines and growth factors in FFA/LPS induced coculture model between Kupffer cells and hepatocytes. Finally, we reported that lower ROS levels along with suppressed proinflammatory cytokines underpin the beneficial activities of IQ in oxidative stress related inflammation and subsequent lipid metabolic abnormalities in liver.

IQ is isolated and processed from* Cichorium glandulosum boiss et huet* (CG), a traditional Chinese medicine present widely in Aksu region of Xinjiang, China [31]. Traditionally, it has beneficial effects on appetite and digestion. In recent past, the published data on IQ has revealed its actions as an antiatherosclerotic [32] and anti-inflammatory agent [33]. Similar data has been presented recently in which IQ has shown to possess antidiabetic activities [23]. In line with traditional uses our work confirms that IQ possesses remarkable antioxidative and lipid lowering activities in hepatocytes* in vitro*. Furthermore, simvastatin is the commonly used statin in cardiovascular diseases and hepatic lipid disorders [34]. As hepatic lipid metabolic disorders lack the specific therapeutic options, simvastatin have emerged as likely options in hepatic lipid overload [35]. We have compared the lipid lowering properties of IQ with simvastatin as reports have confirmed its effectiveness in hepatic lipids accumulation [36].

FFA is the major factor and key determinant in hepatic lipid metabolism [37]. FFA induced hepatic lipid accumulation has been found in variousconnections with other metabolic diseases independently [38]. In addition to its ability to generate the lipid storage conditions* in vitro*, it has manifested the effectiveness to achieve oxidative stress within hepatocytes which is an indicator of abnormal lipid metabolic state [39]. We have induced the hepatocytes with FFA which was successfully able to produce the fat overload condition along with oxidative stress.

Liver is the center stage for lipid metabolism. The excessive lipid intake through diet is stored predominantly in hepatocytes which has become major reason for various clinical metabolic complications. No doubt hepatocellular lipids overload has become the single most important marker for fat related diseases. Our results have proved that IQ can reverse the FFA induced lipid overload dose dependently within hepatocytes. Moreover, TG is an indicator of lipid overload within hepatocytes and its intracellular level has been observed to increase in all metabolic abnormalities. Our data showed that IQ has reversed the increase in intracellular TG level initiated by FFA.

One of the outcome of steatotic liver is the overproduction of ROS as a result of electron leakage during mitochondrial *β*-oxidation in energy production [1]. Persistent generation of ROS culminates in oxidative stress which been shown to be involved in hepatic lipid dysregulation [40]and seem to prime the liver for further lipid storage. Moreover, subsequent proinflammatory cytokine production by elevated ROS also presented to be involved in liver steatosis and progression into NASH [41]. Our results showed that IQ decreased the ROS level to the normal state at suggesting its notable antioxidative properties. Moreover, IQ enhanced the antioxidant enzyme SOD level within both primary hepatocytes and BRL-3A cells. Similarly level of MDA was decreased dose dependently in hepatocytes after administration of IQ.

To refute the possibility that simultaneous administration of IQ and FFA (mixture of IQ and FFAs in medium) can affect the hepatocytic uptake of FFA and hence is responsible for lipid lowering and antioxidative potentials of IQ, we employed variation in FFA model and opted to administer FFA 24 hours prior to the administration of IQ for another 24 hours. Antioxidative and lipid lowering potentials of IQ were in line with the previously used combined administered (FFA and IQ) model. This shows that there is minimal chemical interaction between IQ and FFA outside the hepatocytes and IQ is responsible for these activities without any obvious intervention from FFAs.

To show whether antioxidative properties of IQ can correlate and coexist with its lipid lowering effects we used lipid overload model (FFA) with enhanced oxidative stress (H_2_O_2_) alone or together. H_2_O_2_ increased the relative lipid storage within primary rat hepatocytes in the presence of FFA as compared to FFA alone, leading to conclusion that ROS contributes to the lipid storage process in hepatocytes (Figure 7). IQ markedly lowered the lipid accumulation induced by FFA/H_2_O_2_. Interestingly, lipid lowering effects of IQ coexisted with its antioxidant effects. As it is expressed in Figure 7, IQ dose dependently reduced the intracellular ROS level induced by FFA/H_2_O_2_ and these effects were matched by its dose dependent lipid lowering effects on the same model. As it is shown in Figure 7 NAC blocked the ROS and its effects were comparable with IQ. However, NAC was not as effective in lowering hepatic lipids in contrast to IQ.

It was surprising, though, that NAC was unable to provide expected lipid control in this model. The role of NAC as a lipid lowering agent is confusing and remains obscure although scores of studies have established it as an excellent antioxidant especially in poststeatotic scenario [42, 43]. Fewer studies have attempted to directly study the lipid lowering behavior of NAC especially in hepatic steatosis and those who did showed little or nonpromising prospects of NAC as a lipid lowering agent [44]. Some studies also went on to explain the beneficial role of NAC in “second hit” of fatty liver disease of oxidative stress without any substantial impacts on lowering lipids. These observations are in line with our* in vitro* study results which have shown its effectiveness as antioxidative agent with nonlinear impacts on hepatic lipids. However, it may be plausible to argue that antioxidative potentials of IQ contributes to its lipid lowering benefits with some other possible lipolytic mechanism involved as well, which has produced synergistic effects unlike NAC. Further research in this area can open exciting prospects for effectiveness and limitations of NAC in hepatic liver diseases.

AMPK is a key lipid regulatory gene involved in liver. Activation of AMPK leads to range of beneficial lipid metabolic changes at molecular level including enhanced lipid transport, activation of key lipolytic genes, and inhibition of lipogenic genes. Upstream it is regulated predominantly by LK*β*1, CaMKK1, and raised AMP: ATP. Excess FFA intake is involved in the inhibition of AMPK, while starvation is the primary mechanism to be involved in the activation of this key protein. Relationship between ROS and AMPK remains unclear as conflicting reports have been put forward. Some studies have shown that ROS can be blocked by the activation of AMPK [45] as part of the metabolic benefits of AMPK. As IQ showed both lipid regulating and antioxidative effects, therefore we suspected that AMPK activation can also be involved at molecular levels. In line with our hypothesis, experiments have demonstrated that IQ has significantly upregulated the gene expression of p-AMPK, and p-ACC inhibited by FFA. Treatment with compound C substantially blocked the activation of AMPK and AICAR or IQ was unable to phosphorylate AMPK in the presence of compound C, suggesting the activities of IQ on AMPK. Additionally, FFA induced mRNA expressions of vital AMPK controlled metabolic genes like SREBP-1c, FAS, and PPAR-*α* were reversed by IQ. CPT-1, a redox sensitive mitochondrial gene, was observed to be enhanced by IQ, stressing the involvement of antioxidative potential in metabolic benefits.

Kupffer cell forms the basic defense mechanism in liver. Interestingly, activation of Kupffer cells has been widely reported to generate elevated levels of ROS [6] and produce proinflammatory cytokines such as IL-6, IL-1*β*, TNF-*α*, and TGF-*β*. It has become clear now that these Kupffer cells generated proinflammatory cytokines are involved in the lipid metabolic dysregulation within hepatocytes. Indeed, depletion of KCs has resulted in the metabolic benefits [46] and curbed the oxidative stress in hepatocytes. It may be rational to argue those detrimental metabolic effects of Kupffer cells and its induced proinflammatory cytokines are partly modulated by enhanced ROS levels which is well acknowledged to interfere in the energy metabolism in liver. To prove the metabolic effects of IQ via oxidative stress and proinflammatory activities, we cocultured KCs and HCs and induced it with combined FFA/LPS model. Further, we performed cytokines array to analyze 34 major cytokines released from KCs and detected in the HCs supernatant.

Interleukins (IL) family is the group of cytokines that initiates cascade of at molecular levels. We analyzed the major proinflammatory members of IL family. IL-6 [47] and IL-1*β* [48] are major proinflammatory cytokines that have been linked with the hepatic metabolic dysregulation. Other members of IL group also possess sufficient evidences of lipid metabolic intervention. Moreover, generation of oxidative stress is one of the prominent features of the IL family in hepatocytes. In line with the previous studies, we found that all the major members of IL family were upregulated by FFA/LPS and IQ treated groups drastically suppressed them. Additionally, harmful effects of TNF-*α* on hepato-lipo metabolic system are broadly understood. Expectedly, FFA/LPS induced KCs produced higher levels of TNF-*α* in supernatant of HCs, while IQ treated groups notably reversed these changes.

Furthermore, FFA/LPS induced coculture between HCs and KCs also produced greater levels of ROS, which is in accordance with the previous data that proinflammatory cytokines induce oxidative stress within hepatocytes. Intriguingly, IQ curbed the ROS levels to normal state at 50 *μ*M.

In summary, we have provided the lipid metabolic connections of antioxidative activities of IQ in FFA induced lipid overload and proinflammatory insult in rat hepatocytes. Lack of animal and clinical data is a limitation of our work but this study can provide important blue prints for future research in this direction.

## 5. Conclusion

In conclusion, we proved that IQ contains robust antioxidative and anti-inflammatory potentialities in fat induced hepatocytes and that these activities may partly be responsible for the lipid lowering activities of IQ. Furthermore, AMPK pathway is implicated at the molecular levels for regulation of hepatic lipids by IQ.
